# Kinematic Changes in the Uninjured Limb After a Traumatic Brachial Plexus Injury

**DOI:** 10.3389/fnhum.2021.777776

**Published:** 2021-12-09

**Authors:** Lidiane Souza, Luiggi Lustosa, Ana Elisa Lemos Silva, José Vicente Martins, Thierry Pozzo, Claudia D. Vargas

**Affiliations:** ^1^Laboratório de Neurobiologia do Movimento, Instituto de Biofísica Carlos Chagas Filho – Universidade Federal Rio de Janeiro, Rio de Janeiro, Brazil; ^2^Núcleo de Pesquisa em Neurociências e Reabilitação, Instituto de Neurologia Deolindo Couto – Universidade Federal Rio de Janeiro, Rio de Janeiro, Brazil; ^3^INSERM UMR 1093-CAPS, Université Bourgogne Franche-Comté, UFR des Sciences du Sport, Dijon, France

**Keywords:** kinematic analysis, motor planning, brachial plexus, uninjured limb, upper limb, peripheral nerve injury

## Abstract

**Background:** Traumatic brachial plexus injury (TBPI) typically causes sensory, motor and autonomic deficits of the affected upper limb. Recent studies have suggested that a unilateral TBPI can also affect the cortical representations associated to the uninjured limb.

**Objective:** To investigate the kinematic features of the uninjured upper limb in participants with TBPI.

**Methods:** Eleven participants with unilateral TBPI and twelve healthy controls matched in gender, age and anthropometric characteristics were recruited. Kinematic parameters collected from the index finger marker were measured while participants performed a free-endpoint whole-body reaching task and a cup-to-mouth task with the uninjured upper limb in a standing position.

**Results:** For the whole-body reaching task, lower time to peak velocity (*p* = 0.01), lower peak of velocity (*p* = 0.003), greater movement duration (*p* = 0.04) and shorter trajectory length (*p* = 0.01) were observed in the TBPI group compared to the control group. For the cup-to-mouth task, only a lower time to peak velocity was found for the TBPI group compared to the control group (*p* = 0.02). Interestingly, no differences between groups were observed for the finger endpoint height parameter in either of the tasks. Taken together, these results suggest that TBPI leads to a higher cost for motor planning when it comes to movements of the uninjured limb as compared to healthy participants. This cost is even higher in a task with a greater postural balance challenge.

**Conclusion:** This study expands the current knowledge on bilateral sensorimotor alterations after unilateral TBPI and should guide rehabilitation after a peripheral injury.

## Introduction

The brachial plexus comprises a dense network of spinal nerves originating from vertebrae C5 to T1. Traumatic brachial plexus injury (TBPI) is more commonly found in young adults involved in motorcycle accidents (Faglioni et al., 2014), and typically causes sensory, motor and autonomic deficits of the affected upper limb (Resnick, 1995). The injury can partially or entirely affect the brachial plexus nerve roots (C5-T1) (Dubuisson and Kline, 2002; Moran et al., 2005). As a consequence, proximal shoulder and elbow flexor muscles are those most susceptible to paralysis and sensory loss (Özkan and Aydin, 2001), with the degree of sensorimotor dysfunction varying as a function of the lesion extent and severity (Crouch et al., 2016). Although complete reconstruction of the damaged peripheral nerve pathways is not possible, complex reconstructive surgeries (Noland et al., 2019) and physical therapy (Kinlaw, 2005; Milicin and Sîrbu, 2018; Rich et al., 2019; Chagas et al., 2021) are often performed to restore the motor function of the affected upper limb.

Employing posturographic measurement, Souza et al. (2016) found that TBPI affects body balance, suggesting that the consequences of TBPI on motor control are not restricted to the injured upper limb. Webber et al. (2019) showed that TBPI patients display a greater range of motion of the trunk accompanied by limited shoulder external rotation while performing activities of daily living with the injured arm. In a similar way, Nazarahari et al. (2020) found increased trunk motion when the injured shoulder was performing flexion/extension and abduction/adduction as compared with the uninjured side. These results point toward plastic modifications of the motor plan after a TBPI in respect of upper limb movements, presenting a potential challenge to postural balance.

In another line of evidence, several studies have demonstrated that TBPI is capable of promoting structural and functional modifications in the sensory (S1) and motor (M1) primary cortices contralateral and ipsilateral to the affected side (Mano et al., 1995; Malessy et al., 1998; Beaulieu et al., 2006; Anastakis et al., 2008; Taylor et al., 2009; Davis et al., 2011; Jaggi and Singh, 2011; Yoshikawa et al., 2012; Liu et al., 2013; Fraiman et al., 2016; Lu et al., 2016; Amini et al., 2018; Torres et al., 2018; Fischmeister et al., 2020). Liu et al. (2013) observed changes in interhemispheric connectivity between motor areas, while Fraiman et al. (2016) observed reduced functional connectivity in the representation of the trunk and upper limbs bilaterally in M1, suggesting that TBPI might result in alterations of motor control beyond the affected limb. Recently, Rangel et al. (2021) investigated the occurrence of prediction markers in anticipation of observed sensorimotor events in individuals with upper trunk TBPI. The results showed that TBPI specifically affected the ability to predict upcoming tactile events for the dominant limb. Furthermore, TBPI blurred the prediction markers of upcoming movements in the sensorimotor cortex contralateral to the uninjured limb, indicating that higher order plastic effects might occur following a peripheral sensorimotor loss. This evidence therefore suggests that kinematic changes in the uninjured limb occur after a TBPI.

Kinematic parameters of goal-directed actions have long been shown to reflect their motor plan (Bernstein, 1967; Soechting and Lacquaniti, 1981; Marteniuk et al., 1987; Papaxanthis et al., 1998; Desmurget et al., 1999; Svoboda and Li, 2018). The motor plan encodes where the reach will land on average (the endpoint) and the expected movement duration (Wolpert and Landy, 2012). Several studies have shown that reaching movements display regularities such as typical straight trajectories and bell-shaped velocity profiles (Bernstein, 1967; Atkeson and Hollerbach, 1985; Flash and Hogan, 1985; Marteniuk et al., 1987; Soechting and Flanders, 1991). Duration is an important kinematic component because of the speed-accuracy tradeoff, with movements that take more time to execute being spatially more accurate (Wolpert and Landy, 2012). In addition, Marteniuk et al. (1987) showed that when the task demands greater precision (grasping versus pointing, for example), the duration of the deceleration phase of the trajectory is increased as a consequence of the greater demand for sensory feedback to perform the task.

Motion analysis after TBPI has been used in the clinical context in order to quantify compensatory trunk movements and shoulder dysfunction, and thus help prioritize secondary surgical targets (Webber et al., 2019; Nazarahari et al., 2020). Webber et al. (2019) identified compensatory trunk movements accompanied by limited external rotation of the shoulder when individuals with TBPI performed feeding and dressing tasks. The authors concluded that the restoration of external rotation of the shoulder would be a beneficial secondary target of surgical recovery of motor function. In addition to being a tool for objective outcome evaluation of TBPI and its biomechanical consequences, motion analysis also allows inferences about motor planning aspects to be made through calculation of spatiotemporal variables. Kinematic analysis can thus be profitably used to explore further changes in motor planning after a TBPI. Since bilateral alterations in the sensorimotor cortex have been reported to occur after a TBPI (Liu et al., 2013; Fraiman et al., 2016; Ramalho et al., 2019) our conjecture is that an unilateral TBPI might lead to changes in the motor plan of both limbs. To our knowledge no investigation has focused specifically on the kinematics of the uninjured limb. Measuring the kinematics of the uninjured limb might reveal changes in the motor plan of the upper limbs induced by TBPI and help to guide rehabilitation programs.

The main objective of this cross-sectional study was to investigate the kinematic features of the uninjured upper limb in participants with TBPI. Specifically, we analyzed the kinematic parameters of movement performed with the uninjured upper limb of individuals with TBPI in a free-endpoint whole-body reaching task. This task allows the subjects to freely choose their final hand position, introducing a spatial ambiguity and exposing the subject to a number of subjective choices (Haggard, 2008; Andersen and Cui, 2009; Berret et al., 2011; Hilt et al., 2016). Furthermore, this task presents a postural challenge that allows the investigation of the individual strategies used to choose a suitable hand trajectory toward the target while conserving postural balance (Flanders et al., 1999; Hilt et al., 2016). In addition, we introduced a new task (bringing a cup to the mouth) to investigate the association between the postural component and motor planning in individuals with TBPI, as this task requires minimal trunk displacement. We hypothesized that kinematic features of the uninjured upper limb would be affected by a unilateral TBPI as compared to healthy individuals.

## Materials and Methods

### Participants

Patients with TBPI were recruited from a database (Patroclo et al., 2019) maintained by the Laboratory of Neuroscience and Rehabilitation of the Institute of Neurology Deolindo Couto of the Federal University of Rio de Janeiro from June 2018 to September 2019. This database contains patients’ epidemiological, physical and clinical information collected by a multidisciplinary team through digital questionnaires. The following inclusion criteria were applied: age between 18 and 50 years, right-handed (Oldfield, 1971) before the lesion and with unilateral TBPI, diagnosed by clinical evaluation and/or complementary exams. Individuals were excluded if they presented other neurological injuries, a Mini-Mental State Exam (Folstein et al., 1975; Brucki et al., 2003) score below 24, visual loss or uncorrected visual impairment. Upper extremity function after a TBPI was measured using the version of the Disabilities of the Arm, Shoulder and Hand questionnaire (DASH) adapted to Brazilian Portuguese (Orfale et al., 2005). The DASH is composed of 30 questions that address the ability to perform upper extremity activities and the severity of symptoms (Hudak et al., 1996). Each question is scored on a scale from 1 to 5, with a total score rate from 0 to 100. A higher score reflects greater disability.

All participants were informed about the experimental procedures and provided a written consent form before the tests. The procedures were approved by the ethics committee of the Institute of Neurology Deolindo Couto, the Federal University of Rio de Janeiro (process number: 1.375.64).

### Experimental Procedure

#### Whole-Body Reaching Task

**FIGURE 1:**
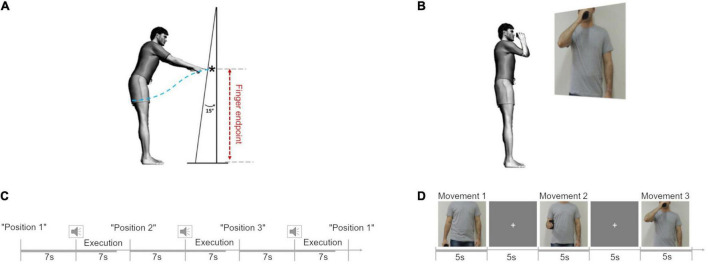
Experimental protocol. **(A)** Movement trajectory length (blue dotted line) and finger endpoint (black star) during the reaching in position 1 (P1). Adapted from Hilt et al. (2016). **(B)** Cup-to-mouth task. **(C)** Schematic representation of the fixed stimuli sequence in the reaching task. **(D)** Schematic representation of the sequence of stimuli in the cup-to-mouth task.

The experimental protocol was adapted from the study by Hilt et al. (2016). From a standing position, the participants were asked to perform a series of reaching movements toward a homogenous surface upon which no specific endpoint was drawn. This surface (2.0 m high × 0.9 m wide) consisted of a white plastic screen positioned at an angle of 15° from the vertical, located at a distance of 120% of the arm’s length from the surface of their shoulder (measured from the acromion to the apex of the index finger). The distance and angle were chosen to allow a significant reaching distance, requiring the controlled maintenance of equilibrium without placing subjects beyond the limits of their balance (Figure 1A; Hilt et al., 2016). Thus, changes in the kinematic metrics could be attributed to the combined motions of the uninjured limb and trunk. The participants were positioned on a rigid surface while their feet were positioned hip-width apart and parallel to the sagittal plane. Reflective markers (15 mm in diameter) were placed in the apex of the right and the left index fingers.

Participants were instructed to remain facing forward, without turning their body, and with their arms relaxed at their sides. The task was to point toward the apparatus using their arm (left and right) starting from three different initial positions: position 1 (P1), arm relaxed next to the body; position 2 (P2), elbow flexed at 90° forward from the side of the body; position 3 (P3), elbow flexed with the hand at shoulder height. Starting from each of the initial postures the participant was instructed to point toward the apparatus with their index finger at a comfortable speed when they heard a “beep” (Figure 1C).

Six trials (2 per position) were performed as training and the data were not included in the analysis. The reaching tasks were separated into blocks: two blocks were performed with the right upper limb and two blocks were performed with the left upper limb. For TBPI participants, the first two blocks were performed with the uninjured limb, and the last two blocks with the injured limb. Each block contained 24 trials (8 for each start position), totaling 96 trials for each participant.

The initial position sequence could be fixed or variable within a block. We defined as a fixed sequence the consecutive order: P1, P2, and P3. In the variable sequence there was a 20% chance of P3 being altered by P2. The participants performed the task in the following order: 1 block of fixed sequence and 1 block of a variable sequence. TBPI patients only performed the task with their injured upper limb if they were able to adopt and maintain the initial positions. To avoid muscle fatigue participants had intervals of 5 mins between blocks.

#### The Cup-to-Mouth Task

We performed a supplementary experiment to investigate if a simple plastic cup-to-mouth task in a standing position might also cause changes in the kinematic profile in individuals with TBPI. From a standing position, with both arms relaxed by their sides and holding a plastic cup in the moving hand, the participants were asked to perform a series of three different tasks illustrated by showing them a picture projected (Epson PowerLit S18+^®^, Epson, Japan) onto a white wall of an actor doing the following tasks: task 1, keep the arm straight close to the body while holding the cup; task 2, flex the elbow at 90° while holding the cup without any pause in the movement and return to the position in task 1; and task 3, bring the cup to the mouth and immediately return to the position in task 1 (Figure 1B). The picture illustrating the task was shown to the participant for 5 s, in which time they were told to perform the task. They then had to wait for the next picture, which was shown after the exhibition of a fixation cross for 5 s (Figure 1D).

These tasks were also separated into two blocks performed with the right/uninjured upper limb and two blocks performed with the left/injured upper limb for both sequence conditions. Each block contained 24 trials (8 for each task), totaling 96 trials for each participant. Only the data collected in task 3 will be considered in this study, as our goal was to compare the kinematic results of this task with those found in the whole-body reaching task.

### Data Acquisition and Analysis

Movements in three axes (mediolateral, X, antero-posterior, Y and vertical, Z) were recorded at 100 Hz using Vicon Nexus software version 2.2 (Vicon Motion Systems Ltd, Vicon, United States) and a seven-camera motion capture system with 1 Megapixel resolution (Vicon Bonita 10, Vicon, United States). The triaxial coordinate time series of the index finger reflective markers were recorded. The sequence of initial positions was generated using the software Presentation^®^ (Neurobehavioral System, United States). Data acquisition was synchronized with the stimulus presentation through a synchronizer (Vicon Lock).

Pre-processing was performed using Vicon Nexus 2.2 software. The index finger marker triaxial coordinates were reconstructed and analyzed offline using Matlab software (R2015a, Mathworks Inc., Natick, MA, United States). As the index finger describes a curved trajectory, we estimated index finger speed by its tangential velocity ($\vec{v}$) in relation to the path. For each instant of time, the tangential velocity ($\vec{v}\,\left(t\right)$) is calculated by multiplying the sampling frequency (*f*_s_) to the magnitude of index finger displacement vector ($\left|\text{Δ}\,\vec{r}\right|$), which corresponds to the index finger displacement in 3-D space.


$$\vec{v}\,\left(t\right)=\left|\Delta \,\vec{r}\right|\cdot f_{s}$$

The 3-D reconstruction of the tangential velocity profile was estimated and subsequently filtered with a 5th-order low pass filter at 10 Hz cutoff. The movement was divided in two phases: (I) from the initial position to the target (apparatus or mouth) and (II) returning from the target to initial position. The onset of each phase was calculated using 5% of peak velocity (PV) as a threshold value. The same threshold was used to detect the end of each phase, i.e., when the tangential velocity dropped below the 5% of peak velocity (Esteves et al., 2016). Peaks and valleys were detected and marked along the velocity profile of each trial, and each phase of the movement consisted of two valleys and a peak. Phase I was separated for analysis and the tangential velocity profile was time-normalized by a linear interpolation of 200 points. Discrepant velocity profile shapes in each trial for each condition were detected through visual inspection, marked and excluded from the analysis.

#### Outcome Measures

The following dependent variables were calculated for the index finger marker:

• **Vertical Finger Endpoint (VFE)**: index finger endpoint measured on the vertical axis normalized by the height of the participant.
• **Horizontal Finger Endpoint (HFE)**: index finger endpoint measured on the horizontal axis normalized by the upper limb length.
• **Movement Duration (MD)**: the time interval between the onset and offset of the reach movement.
• **Trajectory Length (TL)**: the distance traveled by the index finger marker during the reaching movement.
• **Peak Velocity (PV)**: the maximal velocity attained for each participant in the reaching movement.
• **Time to Peak Velocity (TPV)**: the ratio between the acceleration duration and movement duration. A TPV index greater than 0.5 indicates that acceleration duration was longer than the deceleration duration for the reach movement; conversely, a TPV index less than 0.5 indicates that deceleration duration was longer than the acceleration duration for the reach movement. TPV is the kinematic parameter of the movement considered as providing optimal information about motor plans (Marteniuk et al., 1987; Sartori et al., 2011).

### Statistical Analysis

All the analyzed kinematic variables displayed normal distribution (Kolmogorov-Smirnov calculated *p* ≤ 0.05). A paired *T* test was performed to compare the kinematic parameters collected from the right versus the left upper limb in the control group. No significant difference was observed (*p* > 0.05). Therefore, the kinematic values obtained for each arm (i.e., left and right) were averaged per control subject to perform statistical analysis. Kinematic analysis of the TBPI group corresponds to the uninjured upper limb recording, since only three participants succeeded in performing the tasks with the injured limb. A Mann Whitney *U* test was applied by comparing the kinematic measures of left and right uninjured sides to verify the existence of injury side effects.

A three-way mixed model ANOVA was performed with event sequence (fixed sequence vs. variable sequence), initial position (P1 vs. P2 vs. P3) and group (TBPI vs. control) as independent variables for each kinematic parameter. The level of significance was set at 5%. No main effects were observed for event sequence, so they were removed from the model, leaving a two-way model with the other two factors. A two-way mixed model ANOVA was applied with group and initial position as factors. Tukey’s post-hoc analysis was subsequently applied. All statistical analyses were performed using STATISTICA version 10 (Statsoft Inc., United States). Post-hoc power analyses were conducted using G*Power software (Erdfelder et al., 1996), with α = 0.05, two-tailed, to check the statistical power of our results.

## Results

### Participants

From an initial group of 12 participants with TBPI, 11 individuals (2 females) matched the pre-established criteria. The TBPI group exhibited the following characteristics: median age, 35 years of age (range 22–43); median height 174 cm (range 152–184); and median weight 75 kg (range 39–105). Four participants had left arm and seven right arm TBPI (Table 1). A control group of 12 healthy individuals matched in gender, age and physical characteristics was recruited for the study with the following characteristics: median age, 28 years of age (range 19–48); median height 178 (153–190); and median weight 77 kg (52–101). Anthropometric measures and age were similar between the groups (Mann-Whitney tests *p* > 0.05 for all comparisons).

**TABLE 1:** Traumatic brachial plexus injury (TBPI) individual characteristics.

ID	Age	T1	Injured side	Diagnosis	T2	T3	Surgical Procedure	DASH
TBPI01	37	14	Left	C5–C7	5/8	9/6	Ac-SE/Oberlin	50.8
TBPI02	28	35	Left	C5–C7	3	32	Oberlin	27.5
TBPI03	42	41	Left	C5–C8, T1	3	38	INT–MSC, Ac–SE	43.3
TBPI04	35	51	Right	C5–C7	3	48	Ac–SE, Oberlin	59.5
TBPI05	39	54	Right	C5–C8, T1	11/13	43/41	INT–MSC/Ac–SE	30.0
TBPI06	27	27	Right	C5–C7	15	12	Ac–SE	–
TBPI07	38	116	Right	C5–C8, T1	6/15	110/101	INT–MSC/Ac–SE	59.2
TBPI08	43	53	Right	C5–C6	5	48	Ph-SE, Oberlin, and Tr-Ax	45.0
TBPI09	30	48	Right	C5–C8, T1	6	42	Ac-MSC	45.7
TBPI10	29	70	Left	C5–C7	5	65	Ac-SE and Oberlin	25.0
TBPI11	20	4	Right	Posterior Cord	–	–	None	–

*Age in years; T1, time between the injury and the kinematic assessment in months; T2, time between the injury and the surgery in months; T3, time between the surgery and the kinematic assessment in months; Oberlin, ulnar nerve transfer to musculocutaneous nerve; INT–MSC, intercostal nerve transfer to musculocutaneous nerve; Ac–SE, accessory nerve transfer to suprascapular nerve; Ph-SE, phrenic nerve transfer to suprascapular nerve; Tr-Ax, medial triceps transfer to axillary nerve; Ac-MSC, accessory nerve transfer to musculocutaneous nerve; Disabilities of the Arm, Shoulder and Hand (DASH) was collected in 9 of the 11 participants.*

The characteristics of the individuals with TBPI are presented in Table 1. From eleven participants included in the TBPI group, four had complete (C5–T1) and seven had partial TBPI. Ten participants with TBPI had undergone surgical reconstruction of the brachial plexus and only one had not undergone any surgical procedure. The median time elapsed from the injury to participation in the experimental protocol was 48 (range: 4–116) months. The median DASH score was 45.0 (range: 25.0–59.5; Table 1). Only three individuals in the TBPI group were able to perform the tasks with the injured arm (data not analyzed).

### Kinematic Parameters for Whole-Body Reaching Task

The kinematic analysis of the uninjured upper limb of the TBPI vs. control participants was evaluated by means of a two-way ANOVA. This analysis was performed with group (TBPI patients vs. control) and initial position (P1, P2, and P3) as independent variables for each kinematic parameter. The group comparison results are shown for the tangential velocity profile, movement duration, trajectory length and index finger end-point parameters.

#### Peak Velocity

**FIGURE 2:**
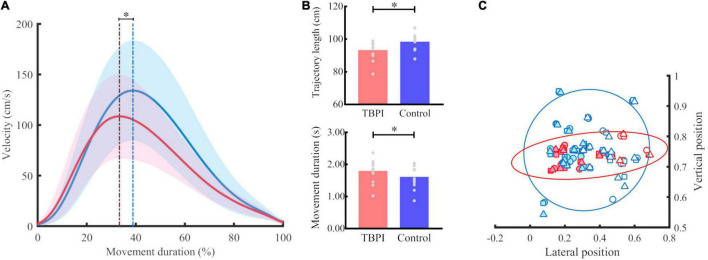
Kinematic performance for groups. **(A)** Tangential velocity profile. Mean (continuous line) and standard deviation (shaded area) for the TBPI group in red and the control group in blue in the reaching phase. The blue dotted line indicates the peak of velocity of the control group and the red dotted line marks the peak velocity of the TBPI group. *p < 0.05. **(B)** Trajectory length and movement duration for TBPI (red bar) and control (blue bar) groups. The average of trials per subject is displayed as a gray dot. *p < 0.05. **(C)** Normalized finger endpoint in vertical and horizontal planes. The symbols represent the normalized average position of the index finger at the end of reaching per subject (TBPI in red, and control in blue) and per starting position (position 1 – circle, position 2 – square, position 3 – triangle). Left finger endpoints are plotted with colored dots, and the right ones with white dots. No significant differences between TBPI and control groups were found for normalized finger endpoint in vertical (*p* = 0.99) and horizontal planes (*p* = 1.00). The covariance data are shown through the 95% confidence ellipse for each group (TBPI – smallest diameter: 0.15, largest diameter: 0.90; Controls – smallest diameter: 0.40; largest diameter: 0.72).

Peak velocity was significantly lower for the TBPI group when compared to the control group [*F*(1, 63) = 9.75, *p* = 0.003; Figure 2A and Table 2]. Moreover, as expected, the PV differed in respect of the initial position [*F*(2, 63) = 25.69, *p* < 0.001; Table 2]. Post-hoc comparisons showed that all positions differed (*p* < 0.01). No significant interaction between group and initial position was found [*F*(2, 63) = 0.1, *p* = 0.91; Table 2]. With our sample size and effect size estimate for the PV variable, the post-hoc power analysis revealed a 51% power to detect a large effect size (*d* = 0.87) between groups.

**TABLE 2:** Mean and standard deviation of the TBPI and control groups per initial position in the reaching task.

Kinematic parameters	TBPI group (*n* = 11)			Control group (*n* = 12)		
	P1	P2	P3	P1	P2	P3
Movement duration (s)	1.78 (0.40)	1.77 (0.36)	1.86 (0.37)	1.56 (0.35)	1.59 (0.35)	1.62 (0.36)
Trajectory length (cm)	111.40 (8.56)	69.27 (5.51)	96.07 (7.68)	120.10 (9.85)	73.67 (5.20)	99.68 (10.62)
Peak velocity (cm/s)	154.20 (36.21)	84.38 (21.80)	120.80 (27.51)	187.60 (40.27)	107.60 (30.22)	149.60 (48.34)
Time to peak velocity	0.30 (0.06)	0.42 (0.06)	0.33 (0.06)	0.37 (0.06)	0.44 (0.06)	0.38 (0.07)
Finger endpoint (V) (%H)	0.74 (0.03)	0.73 (0.03)	0.74 (0.03)	0.75 (0.08)	0.76 (0.08)	0.75 (0.09)
Finger endpoint (H) (%UL)	0.34 (0.18)	0.34 (0.19)	0.34 (0.19)	0.32 (0.15)	0.33 (0.15)	0.33 (0.15)

*Vertical Finger Endpoint (VFE) expressed as a percentage of participant’s height (%H); Horizontal Finger Endpoint (HFE) expressed as a percentage of participant’s upper limb length (%UL).*

#### Time to Peak Velocity

The TPV analysis revealed that the TBPI group had significantly lower values for this parameter than the control group [*F*(1, 63) = 6.64, *p* = 0.01], which means that the duration of the acceleration phase was shorter for the TBPI group when compared to the control group (Figure 2A and Table 2). Differences between the initial position for the TPV parameter were also observed [*F*(2, 63) = 13.83, *p* < 0.001]. Post-hoc analysis showed that P2 differed from the others (P1 and P3, *p* < 0.01; Table 2). No significant interaction between group and initial position was found [*F*(2, 63) = 0.8, *p* = 0.46; Table 2]. With our sample size and effect size estimate for the TPV variable, the post-hoc power analysis revealed a 75% power to detect a large effect size (*d* = 1.17) between groups.

#### Movement Duration

A main effect was found for the group [*F*(1, 63) = 4.38, *p* = 0.04]. The TBPI group showed greater MD compared to the control group (Figure 2B and Table 2). No other effects were observed for this parameter (see Table 2). With our sample size and effect size estimate for the MD variable, the post-hoc power analysis revealed a 27% power to detect a medium effect size (*d* = 0.58) between groups.

#### Trajectory Length

The TBPI group showed significantly shorter TL [*F*(1, 63) = 6.3, *p* = 0.01] compared to the control group (Figure 2B and Table 2). As expected, the TL differed between the initial position [*F*(2,63) = 171.36, *p* < 0.001] for all positions (*p* < 0.01; Table 2). No significant interaction between group and initial position was found [*F*(2, 63) = 0.5, *p* = 0.61; Table 2]. With our sample size and effect size estimate for the TL variable, the post-hoc power analysis revealed a 58% power to detect a large effect size (*d* = 0.94) between groups.

#### Finger Endpoint

No significant differences were found for the FE parameters for both the vertical [*F*(1, 63) = 1.40, *p* = 0.24] and horizontal [*F*(1, 63) = 0.23, *p* = 0.63] planes (Figure 2C and Table 2), although the TBPI participants seemed to exhibit a smaller variability in FE when compared to controls. No differences for initial position comparison were found for the VFE [*F*(2, 63) = 0.01, *p* = 0.99] and HFE [*F*(2, 63) = 0, *p* = 1] parameters (see Table 2).

#### Injury Side Effects

No difference was observed between left and right sides of TBPI except for the TPV parameter in P3 (*U* = 0.00, *p* = 0.003). With our sample size and effect size estimate for the TPV variable in the P3, the post-hoc power analysis revealed a 88% power to detect a large effect size (*d* = 2.25) between groups.

### Kinematic Parameters for the Cup-to-Mouth Task

One-Way ANOVA was applied to compare those same kinematic parameters between the control and the TBPI groups in the cup-to-mouth task (Supplementary Table 2). The TBPI group was composed of the same eleven patients described in Table 1, and the control group was composed of nine out of the same twelve individuals tested in the pointing task. The TBPI group showed lower TPV (0.34 ± 0.04) compared to the control group (0.37 ± 0.02) [*F*(1, 18) = 6.91, *p* = 0.02]. No significant differences between groups were found for PV [*F*(1, 18) = 3.3, *p* = 0.09], MD [*F*(1, 18) = 3.68, *p* = 0.07], TL [*F*(1, 18) = 2.65, *p* = 0.12] and VFE [*F*(1, 18) = 0.12, *p* = 0.73]. With our sample size and effect size estimate for the TPV variable, the post-hoc power analysis revealed a 51% power to detect a large effect size (*d* = 0.95) between groups. No effect of injury side for the cup-to-mouth task was observed for any kinematic parameters.

## Discussion

We aimed to describe the kinematic features of the uninjured upper limb in participants with TBPI. As the free endpoint whole-body reaching task involves a greater postural challenge (Flanders et al., 1999; Hilt et al., 2016) compared to a simple cup-to-mouth task, we hypothesized that the effects of TBPI on the kinematics of the uninjured limb would be more evident in the former than in the latter task. Indeed, whereas lower TPV values were found in the TBPI group compared to the control group for both tasks, lower PV values, greater MD and shorter TLs were found in the TBPI group only for the reaching task.

### Kinematic Changes in the Uninjured Limb: Time to Peak Velocity

For both tasks, a reduced TPV of the uninjured limb was found in the TBPI group compared to the control group. Shorter TPV values translate into a shorter period of acceleration followed by a longer deceleration period while performing the movement. This ratio provides information about the motor strategies used to control actions (Marteniuk et al., 1987; Papaxanthis et al., 1998; Sartori et al., 2011), since the deceleration phase duration increases with the task demand (Marteniuk et al., 1987). Rangel et al. (2021) have suggested that the plastic reorganization after TBPI is associated with modifications in motor planning. Changes in trunk representation, occurring bilaterally in M1 (Fraiman et al., 2016), in addition to balance impairment in adults with TBPI (Souza et al., 2016), could translate as greater motor cost to TBPI participants in performing both tasks in a standing position.

Hilt et al. (2016) showed that in healthy participants reducing the base of support leads to an increased deceleration phase in the TPV parameter. In the same way, the shortening of the acceleration phase and the lengthening of the deceleration phase in the TPV parameter found for both tasks in the present study could relate to the negative impact of TBPI on balance (Souza et al., 2016). A reduced TPV, favoring a lengthened control of the final portion of the reaching movement, could occur as a consequence of postural balance prioritization. Since the reduced TPV was also observed in the TPBI group in the cup-to-mouth task, then the mere control of upright posture could be challenging enough to impact the kinematics of the uninjured upper limb. In fact, although both tasks are performed in a standing posture, there are differences when it comes to their postural challenges.

The two tasks clearly differ in terms of the specific role of the trunk. In the whole-body task, there is a dynamic role for the trunk in assisting the upper limb movement (Hilt et al., 2016), whereas in the cup-to-mouth task the trunk functions rather as a stabilizer, allowing a relative independence of the upper limb movement (Flanders et al., 1999; Hilt et al., 2016). Crucially, the two tasks also clearly differ in the task demand imposed over postural control, being much higher for the whole-body task (Hilt et al., 2016). Thus, the augmented need of postural control imposed by the whole-body task could explain the higher number of altered kinematic parameters observed amongst TBPI participants.

Since a reduced TPV was found for both tasks in the TBPI group, it is reasonable to suppose that the longer deceleration phase would reflect an alteration in the motor plan related more generically to movements of the uninjured limb. Conversely, for the whole-body reaching task there could be a postural balance component associated more specifically with the other kinematic alterations, as discussed below.

### Kinematic Changes in Uninjured Upper Limb: Other Kinematic Parameters

In the whole-body reaching task, the participant has to choose a suitable hand trajectory toward the target while keeping postural balance (Flanders et al., 1999; Hilt et al., 2016). These task constraints can affect both movement planning and execution compared to reaching from a standing position without trunk displacement. Marteniuk et al. (1987) observed that reaching tasks demanding higher precision were executed with longer movement duration and a prolonged deceleration phase (when compared to the acceleration phase). Considering that reaching movements show an initial ballistic phase and a subsequent controlling phase in which fine adjustments are made to successfully achieve the task goal (Desmurget and Grafton, 2000), it is reasonable to think that tasks with higher precision requirements demand more sensory feedback control during the movement deceleration phase (Marteniuk et al., 1987).

Besides the effects of TBPI on the TPV parameter, we found lower PV values and longer MD in the TBPI group compared to the control group when performing the whole-body reaching task. These results are similar to those found by Callegari et al. (2018) who reported that during an upper limb pointing task, individuals in postural positions with less trunk stability showed longer movement duration and lower peak velocity. These effects might indicate greater difficulty in planning upper limb movements (Callegari et al., 2018).

Individuals with TBPI have also exhibited a reduction in TL of the index finger. A reduced trajectory length might reflect detriment in the correlation between trunk and limbs angular displacement (Stapley et al., 1999; Berret et al., 2009). This possibility seems supported by the fact that, although not statistically significant, TPBI end point appeared more compact and more precise than controls, suggesting that the task goal (virtual target to reach) is achieved despite a deterioration of coordination between the arm and the trunk. Moreover, upper limb kinematics was shown to be equilibrium-dependent (Stapley et al., 1999; Berret et al., 2009). For instance, Paizis et al. (2008) found a reduction of wrist trajectory length in young adults in equilibrium restriction conditions similar to those observed in older adult individuals without postural restrictions. The authors proposed that such a strategy becomes dominant in the motor plan of older adults (Paizis et al., 2008). Hilt et al. (2016) observed that the “optimizing balance” strategy was preferred when healthy individuals performed a whole-body-reaching task under reduced postural stability. According to the optimal control theory, motor control will always prioritize a lower cost to achieve the task goal (Todorov, 2004). Our results suggest that the cost to optimize motor planning to achieve the task goal successfully with the uninjured limb was greater for the TBPI group than for the control group.

### Target Specificity

In a free-endpoint whole-body reaching task the participants are free to choose their final hand position, which results in the greater involvement of implicit variables to guide action selection (Haggard, 2008; Andersen and Cui, 2009; Berret et al., 2011; Hilt et al., 2016). Interestingly, participants with TBPI were able to reach the final position as precisely as the control group, independently of the motor strategy used to achieve it. Furthermore, no significant differences were observed between the groups in respect of the finger endpoint parameter in the cup-to-mouth task.

Finger endpoint consistency for both groups, and the differences between the TBPI and control groups for the velocity and trajectory profiles in the reaching task could reflect the motor system’s efficacy in controlling the numerous degrees of freedom involved in the intended movement in order to achieve a same goal (Bernstein, 1967; Turvey et al., 1982; Schöner and Kelso, 1988; Latash et al., 2002; Bizzi et al., 2008). Chang et al. (2009) observed similar results in an animal model of peripheral nerve injury in the ankle extensor muscle; Although there was an alteration in the trajectories of individual joint kinematics, those of limb orientation and limb length remained largely invariant, even when considering the paralyzed ankle extensor muscles. These results suggest that changes in mean joint angles were coordinated as part of a long-term compensation strategy to minimize change in whole limb kinematics (Chang et al., 2009). This strategy may represent a fundamental compensation principle after peripheral injuries that allows the adaptation to the new condition with a minimal effect on overall motor function.

### Differences in Starting Position

The differences observed between the starting positions in the whole-body reaching task for the majority of kinematic parameters confirm that they offer different mechanical constraints for movement planning and execution. Previous studies have shown that the differences in the initial positions of the upper limb in free-endpoint reaching tasks in sitting (Berret et al., 2011) or standing posture (Hilt et al., 2016) did not influence the target choice in healthy subjects. Similar results were observed in our present study, in which a small variability in the target choice was found for both groups independently of the initial mechanical constraints of the upper limb.

### Consequences for Rehabilitation

Current TBPI therapeutic objectives consist of improving muscle strength, range of motion and functionality (Milicin and Sîrbu, 2018; Rich et al., 2019; Chagas et al., 2021), as well as reducing the pain (Lovaglio et al., 2019) of the injured upper limb. Kinematic alterations shown here for the uninjured upper limb in the whole-body task in comparison to the cup-to-mouth task suggest, however, that the greater the postural challenge of the task, the greater is the motor cost to plan and perform it. Indeed, Souza et al. (2016) had already warned about the need to prevent and treat balance impairment in TBPI patients. The present work furthers this proposal by providing evidence in favor of including whole body and uninjured arm movements in TBPI rehabilitation programs. A better understanding of the motor plans changes after a TBPI upon the uninjured limb may help to improve the development of more accurate kinematic measures and more effective and customized rehabilitation programs.

### Study Limitations

The main limitations of the current study were the small sample size as well as its heterogeneity regarding the extent of the injury, the occurrence and type of surgical intervention and the time elapsed since the injury. We have reproduced a very well characterized task to impose trunk displacement (Hilt et al., 2016). This said, a higher number of participants might help to confirm the kinematic results. Also, a direct measurement of the trunk motion would be helpful to identify more precisely its contribution to the altered kinematic parameters found for the uninjured limb as compared to controls.

## Conclusion

This is the first study to show kinematic changes in the uninjured upper limb of adults with TBPI. Our results indicate a higher cost to perform the tasks, reflecting the greater impairment in motor planning and motor control of the individuals with TBPI. These findings expand the current knowledge on bilateral sensorimotor alterations after unilateral TBPI (Liu et al., 2013; Fraiman et al., 2016; Souza et al., 2016; Ramalho et al., 2019). Also, they indicate that treatment after a brachial plexus injury should not be restricted to the affected upper limb and that the postural difficulty of the task should be considered in a treatment program. This study provides important information that could be helpful to guide the rehabilitation program after peripheral injuries. Future research must be performed to better understand the neural mechanisms involved in the kinematic modifications in the uninjured upper limb induced by TBPI.

## Data Availability Statement

The raw data supporting the conclusions of this article will be made available by the authors, without undue reservation.

## Ethics Statement

The studies involving human participants were reviewed and approved by Institute of Neurology Deolindo Couto, Federal University of Rio de Janeiro (process number: 1.375.64). The patients/participants provided their written informed consent to participate in this study.

## Author Contributions

LS, TP, and CV conceived and designed the study. LS and JM recruited and made clinical assessment of participants. LS, LL, and AS collected kinematic data from participants involved in the study. LS and LL organized the database. LS performed the statistical analysis and created manuscript tables. LL and AS designed the figures and legends. LS, AS, LL, and CV wrote the first draft of manuscript. CV and TP provided the mentorship for LS, LL, and AS. All authors gave contributions to different sections in the manuscript, revised, read, and approved the submitted version.

## Conflict of Interest

The authors declare that the research was conducted in the absence of any commercial or financial relationships that could be construed as a potential conflict of interest.

## Publisher’s Note

All claims expressed in this article are solely those of the authors and do not necessarily represent those of their affiliated organizations, or those of the publisher, the editors and the reviewers. Any product that may be evaluated in this article, or claim that may be made by its manufacturer, is not guaranteed or endorsed by the publisher.
