# Supplementary material for: Kinematic Changes in the Uninjured Limb After a Traumatic Brachial Plexus Injury
Source: Front Hum Neurosci. 2021 Dec 9;15:777776. doi: 10.3389/fnhum.2021.777776 (PMC8696281; doi:10.3389/fnhum.2021.777776)
Supplement: Supplementary file 1 [file Data_Sheet_1.docx]

# Supplementary material

| **Table S1:** TBPI patients’ evaluation of upper limb muscles strength by myotome | | | | | | |
| --- | --- | --- | --- | --- | --- | --- |
| ID | C5  (elbow flexors) | C6  (wrist extensors) | C7  (elbow extensors) | C8  (finger flexors) | T1  (finger abductors) | Motor Index |
| TBPI01 | 0 | 3 | 0 | NT | NT | IN |
| TBPI02 | 4 | 2 | 4 | 4 | 2 | 16 |
| TBPI03 | 0 | 0 | 0 | 0 | 0 | 0 |
| TBPI04 | 3 | 5 | 5 | 5 | 5 | 23 |
| TBPI05 | 2 | 0 | 0 | 0 | 0 | 2 |
| TBPI06 | 2 | 0 | 3 | 5 | 3 | 13 |
| TBPI07 | 2 | 2 | 2 | 5 | 2 | 13 |
| TBPI08 | 5 | 5 | 5 | 5 | 5 | 25 |
| TBPI09 | 5 | 0 | 0 | 0 | 0 | 5 |
| TBPI10 | 5 | 5 | 4 | 5 | 3 | 22 |
| TBPI11 | NT | NT | NT | NT | NT | NT |
| Muscular Manual Test: 0- No visible or palpable contraction; 1 - Visible or palpable contraction with no motion; 2 - Full range of movement with gravity eliminated; 3 – Full range of movement against gravity; 4 - Full range of movement against gravity, moderate resistance; 5 - Full range of movement against gravity, maximum resistance. NT – not tested. IN – inconclusive. | | | | | | |

| **Table S2**: Average and standard deviation of the TBPI and control groups in the cup-to-mouth task | | |
| --- | --- | --- |
| Kinematic parameters | TBPI group  (n=11) | Control group  (n=9) |
| Movement duration (s) | 1.21 (0.25) | 1.03 (0.16) |
| Trajectory Length (cm) | 108.02 (10.82) | 115.06 (7.87) |
| Peak Velocity (cm/s) | 189.57 (50.70) | 230.72 (50.01) |
| Time to Peak Velocity | 0.34 (0.04) | 0.37 (0.02) |
| Finger Endpoint (V) (%H) | 0.93 (0.02) | 0.93 (0.02) |
| Vertical (V) finger endpoint expressed as a percentage of participant’s height (%H). | | |
